# Management and Outcomes of Chronic Hepatitis B in Pregnancy: A Retrospective Study from a Tertiary Center in Singapore

**DOI:** 10.7759/cureus.88384

**Published:** 2025-07-20

**Authors:** Huanghuan Li, Sushmitha C, Rajneesh Kumar

**Affiliations:** 1 Gastroenterology and Hepatology, Sengkang General Hospital, Singapore, SGP; 2 Gastroenterology and Hepatology, Singapore General Hospital, Singapore, SGP

**Keywords:** chronic hepatitis b (chb), hepatitis in pregnancy, maternal-fetal transmission, tenofovir alafenamide (taf), tenofovir disoproxil fumarate (tdf)

## Abstract

Introduction

Hepatitis B virus (HBV) infection remains a leading cause of chronic viral hepatitis, particularly in Asia. Vertical transmission during pregnancy is a key contributor to chronic HBV prevalence, and maternal screening with HBV DNA quantification enables the identification of high-risk cases. Antiviral therapy is recommended for pregnant women with HBV DNA levels >200,000 IU/mL to reduce the risk of perinatal transmission. This study aimed to determine the prevalence of high maternal viral load in chronic HBV, assess the initiation of antiviral therapy, and evaluate hepatic flares following treatment cessation postpartum.

Methods

We conducted a retrospective audit of pregnant women with chronic HBV infection referred to a gastroenterology specialist clinic in Singapore from August 2015 to December 2022. Data were collected from electronic medical records. The audit was approved by the institutional board.

Results

Data from 112 patients were analysed. The mean age was 34.6 years (standard deviation (SD) ± 4.4). A total of 25.9% had HBV DNA levels >200,000 IU/mL, with a mean alanine aminotransferase (ALT) of 31.1 U/L, compared to 19.5 U/L in those with lower viral loads. Among high viral load patients, 89.7% (n=26) were started on antiviral therapy; 3 declined treatment. Two patients with a lower viral load but elevated ALT were also treated. Mean gestational age at delivery was 37.7 weeks (SD ± 2.3). Postpartum follow-up revealed 10 cases of hepatitis flare. Eight patients agreed to the re-initiation of treatment. No serious adverse events were reported.

Conclusion

A significant proportion of pregnant women with chronic HBV had high viral loads, necessitating treatment. Tenofovir disoproxil fumarate (TDF) was safe and well-tolerated. Postpartum monitoring is essential due to the risk of hepatic flares following treatment cessation.

## Introduction

Hepatitis B virus (HBV) infection remains a major global public health concern, particularly in Asia, where prevalence rates are among the highest worldwide. According to the World Health Organization, there were 1.5 million new HBV infections, 296 million people living with chronic hepatitis B, and 6 million children under the age of five infected globally in 2019 [1]. That same year, approximately 820,000 individuals died from HBV-related complications, including liver failure and hepatocellular carcinoma [2].

A key driver of HBV persistence is vertical transmission from mother to child. Perinatal transmission alone accounts for up to 50% of chronic HBV infections worldwide. Alarmingly, two-thirds of those infected remain unaware of their HBV status, increasing the likelihood of unintentional transmission in the absence of preventive interventions [3].

Without appropriate prophylaxis, the risk of mother-to-child transmission (MTCT) can reach as high as 90%, especially in mothers who are hepatitis B e-antigen (HBeAg) positive and have high HBV DNA levels. Even with timely infant HBV vaccination within 12 hours of birth alone, MTCT rates in high-risk mothers (HBeAg positive with HBV DNA >200,000 IU/mL) remain between 16-25% [4]. To reduce this risk in such high-risk transmission cases, current perinatal protocols recommend administering both hepatitis B immunoglobulin (HBIG) and the first dose of the hepatitis B vaccine within 12 hours of birth. This is followed by completion of the vaccination series at one and six months. This risk of MTCT is particularly concerning, as it frequently leads to chronic HBV infection in the infants, predisposing them to cirrhosis and hepatocellular carcinoma in the future.

The risk of MTCT strongly correlates with maternal HBV DNA levels. In particular, mothers with viral loads exceeding 6-8 log₁₀ IU/mL face up to a 30% risk of MTCT, even when standard immune-prophylaxis is administered [5]. Accordingly, current guidelines recommend initiating antiviral therapy in the second to third trimester for mothers with HBV DNA levels ≥200,000 IU/mL, typically between 24 and 28 weeks of gestation, to reduce perinatal transmission.

Tenofovir disoproxil fumarate (TDF) is the preferred antiviral agent during pregnancy, given its potent viral suppression, high barrier to resistance, and established safety profile. When combined with the neonatal administration of hepatitis B vaccine and hepatitis B immunoglobulin (HBIG), ideally within 12 hours of birth, this approach significantly lowers the risk of MTCT.

Management of HBV in pregnancy, however, extends beyond the antenatal period. Postpartum hepatic flares are well-documented complications, particularly following the cessation of antiviral therapy. The 2025 European Association for the Study of the Liver guideline recommends the discontinuation of antivirals shortly after delivery in women treated solely to prevent MTCT can be considered, provided there are no other indications for continued therapy [6]. Nevertheless, close postpartum monitoring is essential due to the risk of alanine aminotransferase (ALT) flares, which may necessitate treatment re-initiation.

Despite the availability of effective strategies, optimal HBV management during pregnancy remains complex and continues to evolve. In this context, our study aims to assess the burden of HBV infection among pregnant women at our center in Singapore, focusing on HBV DNA levels and liver enzyme profiles. We also evaluate patterns of antiviral therapy initiation and the extent to which clinical management adheres to established guidelines. Furthermore, we examine pregnancy outcomes and identify any adverse events associated with antiviral therapy. The postpartum course is investigated in detail, with particular attention to the incidence of hepatic flares and the need for the re-initiation of treatment after delivery. Through this study, we seek to contribute the regional data to the expanding body of literature on chronic HBV management in pregnancy. Our findings may help inform clinical practice and enhance the quality of care for pregnant women living with chronic HBV infection.

## Materials and methods

Study design and setting

We conducted a retrospective observational study at a tertiary care center specializing in hepatology and maternal-fetal medicine, serving a diverse urban population in east and central Singapore. The study period spanned from August 2015 to December 2022.

A retrospective design was chosen due to the availability of the electronic medical records spanning several years, allowing for the evaluation of real-world clinical practices and outcomes in a cost-effective and time-efficient manner. This approach enabled us to capture a large sample of patients, reflecting actual clinical decision-making and care over time.

Ethical considerations

This study was conducted as an institutionally approved clinical audit and adhered to ethical standards in accordance with institutional guidelines. Approval was obtained from the Institutional Review Board (SGH 2021-06-00334). Given the retrospective nature of the study, the requirement for individual informed consent was waived. All patient data were anonymized to preserve confidentiality.

Inclusion and exclusion criteria

We included pregnant women aged 18 years and above who were diagnosed with chronic hepatitis B and referred to our center during the study period. Patients were excluded if they had acute hepatitis B infection, co-infection with hepatitis C, hepatitis D, or human immunodeficiency virus (HIV), or if there was evidence of liver cirrhosis or hepatocellular carcinoma (HCC). Cirrhosis and HCC were excluded based on available clinical, biochemical, and imaging data. Specifically, patients with diagnosis codes for cirrhosis or HCC, clinical signs of decompensation (e.g., ascites, variceal bleeding, hepatic encephalopathy), or radiological features suggestive of cirrhosis or liver lesions on liver ultrasound or FibroScan® (where available) were excluded. Blood tests, such as thrombocytopenia and elevated INR, were also considered in conjunction with imaging evaluation.

Data collection

Clinical data were extracted from electronic medical records and encompassed a range of variables, including age, ethnicity, gravidity, and parity. HBV-related parameters, such as HBV DNA levels, HBeAg status, and ALT levels, were also collected. Information on antiviral treatment was obtained, including whether treatment was initiated during pregnancy, the type of antiviral medication used, and documented reasons for treatment refusal. Postpartum follow-up data included the duration of antiviral therapy, ALT levels following treatment cessation, the incidence of hepatic flares, and whether treatment was reinitiated during the postpartum period.

Laboratory methods

HBV DNA levels were quantified using the Roche COBAS® AmpliPrep/COBAS® TaqMan® HBV Test, version 2.0 (Roche, Basel, Switzerland), with a lower limit of detection of 20 IU/mL. ALT levels were measured using automated biochemistry analysers such as the Roche Cobas c502 platform. HBeAg status was assessed using the Abbott ARCHITECT HBeAg chemiluminescent microparticle immunoassay (CMIA) (Abbott Laboratories, Chicago, IL, US).

The upper limit of normal (ULN) for ALT in our institution was 40 U/L. In accordance with the 2025 European Association for the Study of the Liver (EASL) guidelines, ALT elevation is considered clinically significant when above the ULN, especially in combination with HBV DNA levels ≥2,000 IU/mL. In this study, high viral load was defined as HBV DNA ≥200,000 IU/mL, the established threshold for antiviral prophylaxis during pregnancy to prevent mother-to-child transmission.

Treatment protocol

Antiviral therapy with TDF 300 mg once daily was recommended for patients with HBV DNA levels ≥200,000 IU/mL or ALT elevation above the ULN, in combination with HBV DNA levels ≥2,000 IU/mL. Treatment for antiviral prophylaxis was generally initiated between 24 and 28 weeks of gestation and continued until delivery or longer if clinically indicated.

Follow-up protocol

Patients were reviewed every four weeks during pregnancy. Postpartum follow-up occurred at 2, 4, and 12 weeks, with monitoring of HBV DNA and ALT levels. Hepatic flare was defined as an ALT elevation >5 times the ULN or >3 times the baseline value, whichever was greater. In addition, clinical symptoms, such as fatigue, right upper quadrant (RUQ) discomfort, or jaundice, were documented when present. High DNA viral load is defined as HBV DNA levels ≥200,000 IU/mL, and low DNA viral load is defined as HBV DNA levels < 200,000 IU/mL.

Statistical analysis

Descriptive statistics were used to summarize baseline characteristics and outcomes. Continuous variables were expressed as mean ± standard deviation or median with interquartile range, depending on data distribution. Categorical variables were reported as frequencies and percentages.

Comparisons between groups (e.g., high vs. low viral load) were performed using the student’s t-test or Mann-Whitney U test for continuous variables, and chi-square or Fisher’s exact test for categorical variables. A two-tailed p-value <0.05 was considered statistically significant.

All analyses were conducted using SPSS version 25.0 (IBM Corp., Armonk, NY, US).

## Results

A total of 112 pregnant women with chronic hepatitis B were included in the study. The mean age of the cohort was 34.6 ± 4.4 years.

Virological and biochemical profiles

Of the 112 patients, 25.9% (n = 29) had HBV DNA levels ≥200,000 IU/mL, categorizing them as high risk for mother-to-child transmission (MTCT). The remaining 83 patients (74.1%) had HBV DNA levels <200,000 IU/mL. Baseline demographic and clinical characteristics were largely comparable between the two groups (Table 1). There were no statistically significant differences in age, body mass index (BMI), weeks of pregnancy at the time of blood collection, or pregnancy duration.

**Table 1:** Comparison of baseline characteristics of pregnant women by HBV DNA viral load Values presented as mean±SD. HBV, hepatitis B virus; ALT, alanine aminotransferase; AST, aspartate aminotransferase; BMI, body mass index; high viral load, HBV DNA IU/mL; low viral load, HBV DNA<IU/mL

Characteristic	Mean ± SD (Overall)	Mean ± SD (High Viral Load, n=29)	Mean ± SD (Low Viral Load, n=83)	P-value
Age (years)	34.6 ± 4.4	34.6 ± 4.5	34.7 ± 4.3	0.9175
BMI (kg/m²)	24.5 ± 4.5	24.6 ± 4.8	24.6 ± 4.5	1
Pregnancy duration (weeks)	37.7 ± 2.3	37.5 ± 2.0	37.7 ± 2.5	0.6665
Week of pregnancy when bloods were taken	19.5 ± 6.4	20.0 ± 7.5	19.4 ± 6.0	0.6989
ALT (U/L)	22.6 ± 14.6	31.1 ± 21.4	19.5 ± 9.7	0.0082
AST(U/L)	23.7 ± 9.4	28.2 ± 13.2	22.1 ± 6.9	0.0233

However, transaminase levels were significantly higher in the high viral load group. The mean ALT level was 31.1 ± 21.4 U/L in patients with a high viral load compared to 19.5 ± 9.7 U/L in those with low viral load (p = 0.0082). Similarly, aspartate aminotransferase (AST) levels were elevated in the high viral load group (28.2 ± 13.2 vs. 22.1 ± 6.9 U/L, p = 0.0233), suggesting increased hepatic inflammation in this subgroup.

Antiviral therapy initiation

Among the 29 patients with HBV DNA ≥200,000 IU/mL, 25 (86.2%) were initiated on TDF during pregnancy for MTCT prophylaxis. Three patients in this high viral load group declined antiviral therapy due to personal preference. One patient received tenofovir alafenamide (TAF) instead of TDF.

Two patients with HBV DNA levels between 20,000 IU/mL and 200,000 IU/mL were initiated on TDF due to elevated ALT levels. The treatment was based on a diagnosis of immune-active chronic hepatitis B, in accordance with the 2025 EASL guidelines. Both patients had persistently elevated ALT levels >2× ULN and HBV DNA >2,000 IU/mL, fulfilling criteria for immune-active CHB.

Overall, 30 patients (26.8%) received antiviral therapy during pregnancy. Of these, 25 patients were initiated on TDF due to high HBV viral load, while 2 patients received TDF in the context of raised ALT levels. One patient was started on TAF for a high viral load. In addition, two patients were already on antiviral therapy prior to conception and continued treatment throughout pregnancy. A summary of the indications for antiviral initiation and treatment outcomes is presented in Table 2.

**Table 2:** Clinical outcomes of pregnant women with chronic hepatitis B during pregnancy and postpartum HBV, hepatitis B virus; TDF, tenofovir disoproxil fumarate; TAF, tenofovir alafenamide; high viral load, HBV DNA IU/mL; low viral load, HBV DNA<IU/mL; ALT, alanine aminotransferase

Outcome	Number of patients	Percentage of total (N=112)
Patients on antiviral treatment during pregnancy	30	26.80%
Patients with high viral load	29	25.90%
Patients were started on TDF during pregnancy for HBV prophylaxis in view of high viral load	25	22.30%
Patients were started on TDF during pregnancy in view of raised ALT	2	1.80%
Patients who declined antiviral therapy in spite of high viral load due to personal preference	3	2.70%
Patients on TAF for HBV prophylaxis	1	0.90%
Patients on antiviral treatment prior to therapy and continued during pregnancy	2	1.80%
Patients with hepatic flare postpartum	10	8.90%
Patients restarted on treatment postpartum	8	7.10%

Pregnancy outcomes

The mean gestational age at delivery was 37.7 ± 2.3 weeks. There were no serious obstetric complications or treatment-related adverse events reported among patients who received antiviral therapy. 

Postpartum follow-up and hepatic flares

All patients were followed for up to two years postpartum. Of the 26 patients who had received antiviral therapy during pregnancy and subsequently discontinued treatment after delivery, 10 patients (38.5%) developed hepatic flares during the postpartum period. Most flares occurred within the first 12 weeks postpartum. Eight of these patients required a reinitiation of antiviral therapy postpartum, while two patients opted for conservative management with close monitoring. No cases of liver decompensation or fulminant hepatitis were observed during the follow-up period.

**Figure 1:**
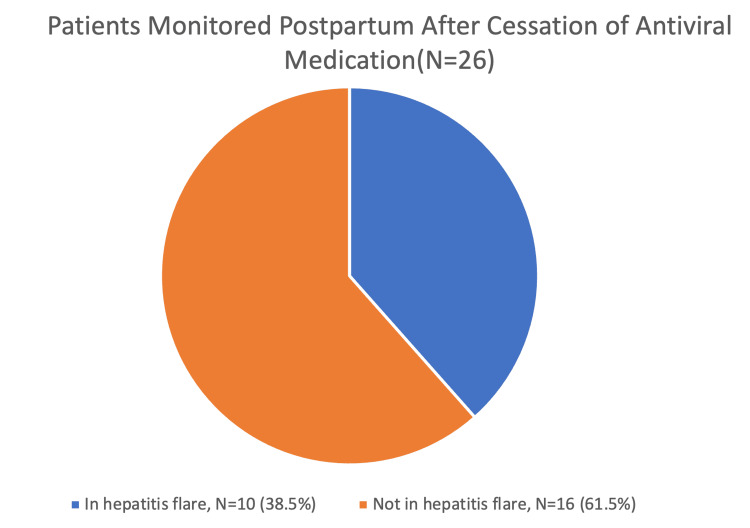
Patients monitored postpartum after cessation of antiviral medication This pie chart illustrates outcomes among 26 patients who discontinued antiviral therapy after delivery. A total of 38.5% (10 patients) experienced a biochemical hepatic flare during the postpartum period.

Figure 1 illustrates the proportion of patients who experienced hepatic flares postpartum during monitoring and the proportion who required a reinitiation of antiviral therapy. Among the 26 patients who had received antenatal antiviral therapy and discontinued it after delivery, 38.5% of them experienced a hepatic flare. This highlights the substantial risk of hepatic flares after treatment cessation and the importance of close postpartum surveillance.

## Discussion

This study describes the clinical management patterns, treatment uptake, and postpartum follow-up of pregnant women with chronic hepatitis B in a tertiary centre in Singapore over a seven-year period. Our findings underscore the importance of stratifying the MTCT risk based on maternal viral load and reaffirm the safety and effectiveness of TDF in reducing perinatal HBV transmission. Additionally, this study highlights the need for vigilant postpartum surveillance following the cessation of nucleos(t)ide analog (NA) therapy due to the risk of hepatitis flares.

MTCT accounts for over 90% of new CHB infections globally, making its prevention central to HBV elimination strategies. The combined administration of hepatitis B vaccine and hepatitis B immunoglobulin (HBIG) within 12 hours of birth has significantly reduced MTCT rates. However, among mothers with high HBV DNA levels (≥200,000 IU/mL), residual transmission risk persists despite immunoprophylaxis. In our cohort, 25.9% of women met this high-risk threshold, and 89.6% of them received antiviral therapy in line with guideline recommendations. The decision of three patients to decline treatment highlights the ongoing need for shared decision-making and patient-centered counselling to reinforce the benefits of MTCT reduction.

While lamivudine and telbivudine have been shown to reduce MTCT, they are no longer first-line therapies due to their lower antiviral potency and low genetic barrier to resistance. In contrast, TDF is preferred due to its superior efficacy and high resistance threshold. In our cohort, patients with high HBV DNA levels had higher mean ALT levels (31.1 U/L vs. 19.5 U/L), suggesting increased liver inflammation. Two patients with HBV DNA between 2,000 and 200,000 IU/mL were restarted on TDF, as they had elevated ALT levels consistent with the immune-active phase of CHB. Specifically, treatment was guided by ALT levels more than the upper limit of normal (ULN). This threshold reflects EASL 2025 recommendations for initiating therapy in non-cirrhotic immune-active patients, where elevated ALT indicates likely progressive liver injury risk [6]. Entecavir is avoided in pregnancy due to limited safety data.

More recently, TAF, a newer tenofovir prodrug, has garnered interest due to its favourable renal and bone safety profile. Although most of the available safety data on TAF use in pregnancy come from studies involving individuals co-infected with HIV and HBV, no significant safety concerns have been reported to date. TAF is now recommended as a first-line agent for pregnant women with HIV [7], and its role in chronic hepatitis B in pregnancy is being explored. A 2024 systematic review and meta-analysis by Pan et al., which included 31 studies, found both TAF (n = 280) and TDF (n = 2,588) to be equally effective in preventing MTCT, with favourable maternal and infant safety outcomes [8]. In our study, the patient treated with TAF during pregnancy experienced no adverse events. These findings align with the 2024 American College of Obstetricians and Gynecologists (ACOG) guidelines, which endorse the use of either TAF 25 mg or TDF 300 mg daily, initiated at 28-32 weeks of gestation, for MTCT prevention.

Pregnancy outcomes in our study were favourable, with a mean gestational age of 37.7 weeks and no significant maternal or neonatal adverse events observed among those who received antiviral therapy. These results support the established safety of TDF in pregnancy. In a landmark randomized controlled trial from Hong Kong, Wong et al. reported MTCT rates of 73.2% without intervention, 21% with vaccination alone, and 6.8% with both vaccination and HBIG in HBeAg-positive mothers [9], underscoring the critical role of immunoprophylaxis in conjunction with maternal antiviral therapy.

Postpartum management remains an essential but often overlooked component of HBV care in pregnancy. Both the American Association for the Study of Liver Diseases (AASLD) (2015) and EASL (2025) guidelines recommend that NA therapy initiated solely for MTCT prevention may be discontinued at delivery or within four weeks postpartum, provided close follow-up is ensured [6,10]. In our cohort, 10 patients (38.5%) developed postpartum ALT flares following therapy cessation, of whom eight required re-initiation of antiviral therapy. Two patients declined retreatment and were closely monitored. Notably, no patients experienced hepatic decompensation, consistent with the 2024 American Gastroenterological Association (AGA) Clinical Practice Update, which emphasizes structured postpartum surveillance [11].

The 38.5% postpartum flare rate observed in our cohort is consistent with previous reports showing that 17.2% to 62% of patients experience hepatic flares following NA withdrawal, most commonly within six months of delivery [12]. In our cohort, all postpartum hepatic flares were clinically mild, resolved without complications. They were not associated with hyperbilirubinemia, hepatic decompensation, or hepatic failure. This was consistent with previous reports describing the typically benign nature of such flares [13]. 

This study contributes valuable local data to the evolving literature on HBV management in pregnancy. Nonetheless, several limitations should be noted. The retrospective nature of our study may introduce selection bias and limit the ability to control for potential confounding variables. As a single-center study, the findings may not be fully generalizable to other institutions or broader populations with different demographic or clinical profiles. Long-term follow-up data beyond 12 weeks postpartum were not consistently available for all patients, which may underestimate the true incidence of delayed hepatic flares or treatment-related outcomes. In addition, infant outcomes, including HBV serologic test results and long-term follow-up, were not consistently captured in the available medical records and were therefore not analysed. These gaps should be addressed in future prospective studies. 

Despite these limitations, this study describes relevant treatment practices, maternal virological profiles, and postpartum monitoring outcomes among pregnant women with chronic hepatitis B managed at a tertiary hepatology referral center in Singapore over a seven-year period. Its strengths include treatment decisions aligned with international guidelines and regular postpartum monitoring. The inclusion of both biochemical and clinical flare characteristics provides granular insights relevant to daily practice. It highlights areas for future research and clinical attention. Future prospective studies with larger cohorts and extended follow-up are needed to address these gaps and better inform clinical practice.

## Conclusions

Our findings support our antiviral therapy for pregnant women with high hepatitis B virus (HBV) DNA levels of ≥200,000 IU/mL during 24-28 weeks of gestation as per international guidelines and demonstrate its safety and effectiveness in clinical practice. Clinicians should initiate tenofovir disoproxil fumarate (TDF) between 24 and 28 weeks of gestation in pregnant women with HBV DNA ≥200,000 IU/mL to reduce the risk of mother-to-child transmission. Postpartum alanine aminotransferase (ALT) flares remain a concern and justify continued close surveillance and monitoring after delivery. Further prospective studies will be beneficial to assess the role of the newer agents, such as tenofovir alafenamide (TAF), in our local context and explore long-term maternal and neonatal outcomes.
